# Gas6/TAM Axis in Sepsis: Time to Consider Its Potential Role as a Therapeutic Target

**DOI:** 10.1155/2019/6156493

**Published:** 2019-08-14

**Authors:** Livia Salmi, Francesco Gavelli, Filippo Patrucco, Marina Caputo, Gian Carlo Avanzi, Luigi Mario Castello

**Affiliations:** ^1^Department of Translational Medicine, University of Piemonte Orientale, Via Solaroli 17, 28100 Novara, Italy; ^2^University of Piemonte Orientale, Via Duomo 6, 13100 Vercelli, Italy

## Abstract

Tyrosine kinase receptors are transmembrane proteins involved in cell signaling and interaction. Among them, the TAM family (composed by Tyro 3, Axl, and Mer) represents a peculiar subgroup with an important role in many physiological and pathological conditions. Despite different mechanisms of activation (e.g., protein S and Galactin-3), TAM action is tightly related to their common ligand, a protein named growth arrest-specific 6 (Gas6). Since the expression of both TAM and Gas6 is widely distributed among tissues, any alteration of one of these components can lead to different pathological conditions. Moreover, as they are indispensable for homeostasis maintenance, in recent years a growing interest has emerged regarding their role in the regulation of the inflammatory process. Due to this involvement, many authors have demonstrated the pivotal role of the Gas6/TAM axis in both sepsis and the sepsis-related inflammatory responses. In this narrative review, we highlight the current knowledge as well as the last discoveries on TAM and Gas6 implication in different clinical conditions, notably in sepsis and septic shock. Lastly, we underline not only the feasible use of Gas6 as a diagnostic and prognostic biomarker in certain systemic acute conditions but also its potential therapeutic role in these life-threatening diseases.

## 1. Brief “TAM” Story

Tyrosine kinase receptors (RTKs) are transmembrane proteins often implicated in cell-to-cell communication. Until now, 58 RTKs have been identified [1]; these receptors pilot, through phosphorylation, an enormous amount of essential signaling pathways, regulating proliferation, survival, and apoptosis.

**Figure 1:**
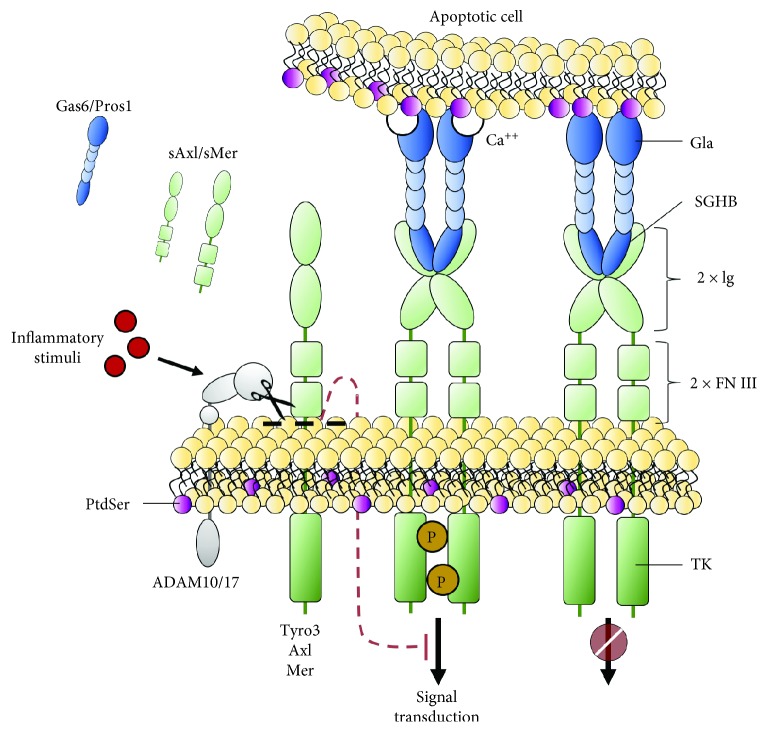
TAM structures and posttranslational regulation. Schematic representation of TAM receptors and their ligands. All TAM receptors share structural domains, i.e., the tyrosine kinase (TK) domain, the transmembrane domain, two fibronectin type III domains (FN III), and two Ig-like domains (Ig) from the C-terminal to the N-terminal (right). The TAM ligands Gas6 and Pros1 share a sex hormone-binding globulin (SHBG) domain and a gamma-carboxyglutamic acid-rich (Gla) domain (right). The Gla domain binds phosphatidylserine (PtdSer) exposed in the outer/external side of the apoptotic cell plasma-membrane, while the SHBG domain interacts with TAM receptor Ig-like domains on the surface of TAM-expressing cells, thus acting as “bridge” proteins (right). The binding itself does not result in receptor activation that occurs through receptor transphosphorylation and in a Ca^++^-dependent fashion (center). For Mer and Axl, the signal transduction is shut down by proteolytic cleavage of the receptor ectodomain (shedding), which is mediated by the transmembrane disintegrin and metalloproteinase (ADAM) 17 and/or ADAM10. Shedding can be induced by inflammatory stimuli (e.g., lipopolysaccharide) leading to the extracellular domain release of the receptor and generating a soluble Axl (sAxl) and soluble Mer (sMer) form able to interact with and sequester the ligands Gas6 and Pros1 (left).

Among RTKs, Tyro3, Axl, and Mer (gene name *Mertk*) share structural similarity (notably two Ig-like domains, two fibronectin type III domains, a hydrophobic transmembrane domain, and a tyrosine kinase domain) and they are grouped in the so-called “TAM family” (Figure 1). Despite their deep resemblance, TAM receptors are expressed by different cell types and tissues (Table 1): Tyro3 is generally localized in the nervous system, whereas Mer and Axl have been found in different tissues and they are frequently coexpressed by the same cells [2]. This coexpression can be either equivalent in some cells, such as Kupffer cells in the liver and red pulp macrophages in the spleen, or unbalanced in others, such as for CD68^+^ tingible macrophages, which are primarily Mer^+^, and CD11c^+^ white pulp dendritic cells (DCs), which are mostly Axl^+^ [3].

TAM were discovered and cloned by several groups in the 90s [2]. In the first years from their discovery, their role in the maintenance of homeostatic balance through the regulation of the phagocytosis of apoptotic bodies (efferocytosis) was demonstrated [21]. Gradually, their role in the innate inflammatory response and in the regulation of cell proliferation and apoptosis was elucidated, leading to growing interest. In fact, a deficiency in TAM expression is related to autoimmunity diseases [2] and, oppositely, their overexpression or aberrant activation (i.e., gain-of-function mutations) is associated with the development and progression of cancer [22].

In this context, the complex network of TAM functions has been clarified in recent years, as it seems more linked to the environmental context, or “*milieu*,” rather than to the expressing cell/tissue, such as neurodegenerative diseases [23], autoimmune diseases, and cancer [24]. TAM activation, which occurs through tyrosine cross-phosphorylation, is normally mediated by the binding with their ligands, growth arrest-specific 6 (Gas6) and protein S (Pros1). Gas6 and Pros1 share in the C-terminal portion the “sex hormone-binding globulin (SHBG) domain,” which binds the TAM Ig-like domains. The N-terminal portion includes the *γ*-carboxylate “gamma-carboxyglutamic acid-rich (Gla) domain,” responsible for binding the phospholipid phosphatidylserine (PtdSer) in a Ca^++^-dependent reaction (Figure 1).

Gas6 is able to bind and activate all TAM receptors, while Pros1 can only bind Mer and Tyro3, without interacting with Axl. In 2014, Lew et al. published a detailed paper showing that Gas6 is capable of binding and activating all TAM, but the most powerful effect was observed following Axl activation. Moreover, both murine and human recombinant Pros1 can bind and activate murine Tyro3 and Mer (but not Axl) in vitro. Lastly, they showed that the PtdSer-binding Gla domain present on Gas6, PtdSer itself, and Ca^++^ are all essential to achieve a full receptor activation, but none of them is involved in receptor binding [25]. Interestingly, Gas6/Pros1-TAM receptor binding is not able to determine the receptor activation per se [25]; so all the conditions described above need to be fulfilled in order to trigger the numerous signal transduction pathways, such as phosphoinositide 3-kinase (PI3K)/Akt, mitogen-activated protein kinase (MAP kinase), nuclear factor-light-chain-enhancer of activated B cells (NF-*κ*B), signal transducer and activator of transcription protein (STAT), phospholipase C (PLC), growth factor receptor-bound protein 2 (Grb2), Raf-1, extracellular-signal-regulated kinase (ERK), and others [26–28]. Rothlin et al. demonstrated that TAM signaling triggers the expression of the suppressor of cytokine signaling proteins, SOCS1 and SOCS3. In fact, in dendritic cells from mice knockout for all three TAM receptors (TAM triple knockout; TAM TKO), the induction of SOCS1 was substantially impaired [29, 30].

Until now, different mutations on TAM receptors have been linked to defined genetic diseases: primarily many MerTK mutations were associated with retinal degenerations [31]. In particular, TAM receptors differ from other RTKs since we know from mouse models that TAM genes can be ablated without any major effect on embryonic development [32]. As a consequence, TAM TKO mice are indistinguishable from their wild-type (WT) counterparts and this aspect appears peculiar because usually the absence of expression of other RTKs leads to severe embryonic development impairment, with intrauterine death [33]. Although during the first three life-weeks no macroscopic difference can be observed between TAM TKO and TAM WT mice, after this period TAM TKO mice develop several degenerative phenotypes. Male TAM TKO mice are infertile in adult life, a condition that is related to impaired sexual development and spermatogenesis. Indeed, Sertoli cells express all three TAM receptors as well as both ligands, Gas6 and Pros1, which allow them to manage, in an autocrine fashion, the phagocytosis of apoptotic germ cells (around 10^8^/day in human male) [34]. The absence of TAM receptors results in incorrect efferocytosis and accumulation of apoptotic cells, damaging sexual organs. Still, both in adult TAM TKO and single Mer^−/−^ mice, the impairment of phagocytosis causes the accumulation of apoptotic debris in the retina, causing a nearly complete absence of photoreceptors [35, 36] and blindness [32].

Since one of the main functions of TAM receptors is to modulate the immune homeostasis [2, 37], it is reasonable to consider their implication in autoimmune phenotypes. Qi et al. have demonstrated that TAM TKO mice develop a spontaneous liver disease which resembles autoimmune hepatitis. These mice exhibited chronic hepatitis, with progressive inflammatory cell infiltration and elevated cytokine levels in the liver [38]. Moreover, TAM TKO mice displayed splenomegaly, lymphadenopathy, and lymphocyte infiltration in nearly all tissues around 4-6 weeks after birth [37]. Also coagulation was impaired with both thrombosis and hemorrhages, especially in the brain, as well as skin lesions and hemophilic-like phenotypes with swollen joints [37].

Additionally, these mice generate high levels of circulating autoantibodies directed against dsDNA, collagens, and phospholipids, such as cardiolipin, PtdSer, phosphatidylethanolamine, and phosphatidylinositol [37].

Thus, we can summarize that TAM TKO mice have an autoimmune phenotype with features comparable to systemic lupus erythematosus (SLE), psoriasis, and rheumatoid arthritis [2, 37]. Antigen-presenting cells (APCs) from TAM TKO mice have a dysregulated activity in response to inflammatory stimuli, demonstrating a reduced tolerogenic behavior with the hyperproduction of type 1 interferons, interleukin (IL) 12, and overexpression of MHC class II and CD86 [29, 37]. This expression pattern is consistent with the splenomegaly and lymphadenopathy observed in adult TAM TKO mice.

Despite their structural homology, following activation TAM receptor signaling is shut down in different ways: the signal desensitization that occurs through the shedding of the ectodomain by proteolytic cleavage was reported for Mer and Axl [39, 40]. In spite of soluble Tyro3 increasing levels in the bloodstream in different chronic diseases [41, 42], this signal desensitization mechanism has not been described for Tyro3 yet (Figure 1). Between the TAM-common-fibronectin type III domains and the transmembrane domain, the proline residue Pro^485^ present in the Mer sequence makes it susceptible to cleavage by the metalloproteinase ADAM17, a disintegrin and metalloproteinase domain 17 [39], also known as tumor necrosis factor-alpha converting enzyme (TACE). Although the examination of the cleavage site sequence of several substrates shed by ADAM17 indicates that the distance between ADAM17 and its target is more important than the specific sequence in ectodomain shedding, the site direct mutagenesis of the Pro^485^ cleavage site results in Mer resistance to proteolysis [39].

The activation of pattern-recognition receptors (PRRs) with lipopolysaccharide (LPS) or polycytidylic acid (Poly:C) in macrophages results in the induction of cleavage of the Mer extracellular domain. Furthermore, LPS- and polyinosinic:polycytidylic acid- (PolyI:C-) induced Mer shedding is dependent on ADAM17, as it is abrogated in ADAM17 gene knockdown macrophages. Sather et al. have shown that the shedding of the Mer ectodomain results in the inactivation of the receptor and in additional neutralization of TAM ligands, which are sequestered by the released soluble form of the receptor ectodomain [43]. This autoregulatory mechanism is not exclusive to Mer but it has been described also for Axl. The cleavage, which generates the soluble and circulating Axl (sAxl), is induced by ADAM17 and another metalloproteinase, ADAM10 [44] (Figure 1). In 2010, Ekman et al. demonstrated that Gas6 is trapped by sAxl. In their elegant study, they hypothesized the absence of free-Gas6 circulating in the bloodstream in healthy subjects, since the molar concentration of sAxl is higher than the one of Gas6, thus suggesting that Gas6 released from cells is quickly bound by sAxl [45]. This seems related to the higher affinity of Gas6 for Axl in comparison to Mer. Indeed, Gas6 binds Axl with a dissociation constant in the subnanomolar range, whereas its affinity for Mer is at least 10-fold lower [46]. So, according to the interpretation suggested by Ekman et al., in the presence of Axl the interaction between Gas6 and Mer or soluble Mer (sMer) might be prevented. Conversely, a previous study published by Sather et al. demonstrated that both sAxl and sMer are able to inhibit the Gas6 activity. The authors focused on sMer, showing that the inactive sMer/Gas6-complex leads to a defective macrophage-mediated engulfment of apoptotic cells. Furthermore, they showed that the release of sMer is associated with a decrease of platelet aggregation in vitro and it could prevent the fatal collagen/epinephrine-induced thromboembolism in mice [43].

## 2. TAM Ligands: Mediators in Cell-to-Cell Interactions

To date, Gas6 and Pros1 are the most known TAM ligands, but other new potential ones have been described: tubby, tubby-like protein 1 [47], and galactin-3 (Gal-3) [48] seem to preferentially activate Mer during phagocytosis. However, little is still known regarding these new TAM ligands and this issue is beyond the scope of this review.

Both Gas6 and Pros1 are members of the vitamin K-dependent protein family: in fact, they contain a Gla domain in which the glutamate residues are posttranslationally modified to form gamma-carboxyglutamate through a vitamin K-dependent carboxylation. This latter reaction is required to confer to these proteins their activities. Moreover, Gas6 and Pros1 contain the SHBG-like domain that makes them unique compared to other vitamin K-dependent proteins: this domain shares 30% sequence identity with SHBG, it replaces the serine-protease domain found in other vitamin K-dependent plasma proteases [49], and it is devoid of enzymatic activity [50].

Pros1 circulates in plasma at a concentration of 346 nmol/L [51], and its expression can be found in several organs, such as the liver, kidney, lungs, and gonads [51], where it is produced by different cell types, like hepatocytes, endothelial cells, megakaryocytes, and osteoblasts [52]. Pros1 heterozygous deficiency is associated with an elevated risk of thrombosis development, whereas homozygous deficiency is lethal during embryonic development [51]. As stated above, Pros1, together with Gas6, is the most studied TAM ligand; it presents ~42% homology sequence with Gas6, and it specifically binds/activates Mer and Tyro3. Although Gas6 and Pros1 share structural homology, their functions are dissimilar, since the functions of Gas6 are limited to binding TAM. Instead, it is important to specify that Pros1 circulates in the bloodstream in two different forms: 60% of Pros1 is bound to the C4b-binding protein, while the remaining 40% of Pros1 is freely circulating [53]. Thus, only the “free Pros1” can bind and activate Mer and Tyro3. In addition, Pros1 contributes to the downregulation of thrombin formation by stimulating the activity as a nonenzymatic cofactor of both activated protein C and tissue factor pathway inhibitor [54, 55]. This latter essential function is TAM independent.

Gas6 interacts with TAM through its SHBG-like domain, positioned at the C-terminus of its sequence, activating downstream signaling pathways, such as PLC*γ*, PI3K, ERK, and NF-*κ*B, and regulating cell survival, proliferation, migration, differentiation, adhesion, and apoptosis [56, 57].

Gas6 expression has been described in CD11b^+^F4/80^+^ bone marrow macrophages [58], in microglia [59], in peritoneal macrophages [14, 60], in apoptotic thymocytes [19], in Sertoli cells [61], and in CD11c^+^ dendritic cells of colon carcinoma [60]. Moreover, Gas6 is particularly expressed by endothelial cells, platelets, and leukocytes [62, 63].

Despite this, the biological role of Gas6 is not completely understood yet. Goruppi et al. showed that Gas6 is able to induce proliferation in vitro and to promote survival in the murine fibroblast cell line NIH-3T3 [64].

During the last years, different groups studying Gas6-TAM interaction focused on inflammation and tissue homeostasis, since in the presence of the Gla domain binding a PtdSer and the SHBG-like domain binding the Ig-like domain of TAM, Gas6 works as a bridge between apoptotic cells and the effector cells (Figure 1).

## 3. Gas6 and TAM Involvement in the Pathophysiology of Different Acute and Chronic Diseases

Gas6 and Pros1 are secreted in the bloodstream and, interestingly, Gas6 plasma levels in humans (~18 ng/mL) are two logarithms lower than Pros1 plasmatic ones [65]. Gas6 expression and its concentration in the bloodstream and in different compartments were found to change in several pathological conditions, both chronic and acute. These data allowed hypothesizing a role for Gas6 in the physiopathology of different diseases and using it as a tool for prognostic stratification in several specific contexts. For example, Bellan et al. demonstrated a correlation between plasmatic Gas6 levels and liver stiffness due to hepatic fibrosis from several etiologies [66]. In this context, they have introduced thresholds of plasmatic Gas6 for liver fibrosis (30 ng/mL) and severe fibrosis (42 ng/mL). Furthermore, the role of Gas6 as a predictor of esophageal varices was esteemed in patients affected by hepatitis C virus-related chronic liver disease [67]. In 2017, Staufer et al. strongly demonstrated the utility of sAxl and Gas6 serum levels as a diagnostic tool for advanced fibrosis, cirrhosis, and hepatocellular carcinoma on 392 patients, 361 of whom were affected by chronic liver disease from different etiologies. Moreover, they suggested the sAxl/albumin ratio as a better biomarker, since this ratio increases the accuracy to detect the degrees of these chronic liver diseases [68]. The use of Gas6 as a noninvasive biomarker has been proposed also by Li et al. in the early detection of diabetic nephropathy [69]. On the contrary, they observed decreased levels of Gas6 in diabetic patients suffering from the underestimated nephropathy and have proposed Gas6 (cutoff~9 ng/mL) as a better biomarker than cystatin C and creatinine. Concerning the renal pathophysiology, it has been shown that not only Gas6 but also sMer and sAxl have a potential role as biomarkers in patients affected by chronic kidney disease (CKD). Monocytes derived from CKD and hemodialysis patients showed a downregulation of Mer and Axl expression, both at RNA and plasma-membrane protein levels. However, plasmatic sMer and sAxl levels were remarkably higher in comparison to healthy subjects and they resulted to be positively associated with Gas6 levels in plasma of CKD patients [70].

Moreover, Sainaghi et al. found high Gas6 levels in the cerebrospinal fluid (CSF) of patients with a diagnosis of Alzheimer's disease (AD), with values that were doubled compared to the control group. The authors justified these findings as a compensatory mechanism: they hypothesized a Gas6 attempt to downregulate the proinflammatory cytokines, which are partially responsible for neuronal death [71]. Additionally, Gas6 has been found poorly expressed in the plasma of patients affected by multiple sclerosis, unlike sMer and sAxl [72]. However, Gas6 levels were found higher in CSF of these patients compared with control group, correlating with the relapse severity of the disease [73, 74]. Gas6 protein concentration in CSF was also found elevated in patients with chronic inflammatory demyelinating polyneuropathy (CIDP) [75].

The Gas6 role as biomarker in SLE patients, particularly for those developing lupus nephritis and cutaneous vasculitis, suggested by Wu et al. in 2014 [76], has been recently confirmed by Gong et al. [77]. In addition, they showed an increase in the levels of soluble forms of Mer and Axl in these patients and they correlated the high levels of soluble receptors to proliferative glomerulonephritis.

However, the association between autoimmune diseases, SLE, and (s)TAM level/role is well established and reviewed elsewhere [24, 78].

Since TAM and their ligands have a wide range of functions and are expressed all over the body, it is reasonable to think of their possible involvement in acute diseases as well. It is reported that plasma Gas6 concentration is increased in patients with acute dyspnea due to heart failure and even more in patients with systemic or pulmonary infection [79]. Llacuna et al., for example, assumed a feasible therapeutic role of Gas6 after ischemia/reperfusion- (I/R-) induced hepatic injury in mice. They demonstrated that Gas6 homeostasis is regulated during I/R with its local release aimed at plugging the injury during the first phase; then, they observed a drastic decrease of Gas6 RNA during the reperfusion phase. Using mice knockout for Gas6 (Gas6^−/−^), the authors highlighted an increased susceptibility to hepatic I/R injury associated to enhanced expression of proinflammatory cytokines, such as IL-1*β* and tumor necrosis factor *α* (TNF-*α*), and increased levels of hepatic transaminases (alanine aminotransferase (ALT) and aspartate aminotransferase (AST)). Moreover, they intravenously injected recombinant Gas6 (rGas6) in mice after hepatic I/R, in both Gas6 WT and Gas6^−/−^ mice, observing that rGas6 injection not only rescued null mice from I/R-mediated liver injury but it also proved to be useful in protecting WT mice against hepatic I/R damage [80].

The therapeutic role of Gas6 has been suggested also by two other research groups using mouse models of sepsis-induced kidney injury [81] and sepsis-induced lung injury [82]. Chen et al. reported that intravenous injection of rGas6 immediately after sepsis induction exerts protective effects by reducing serum urea nitrogen, creatinine, and renal tissue apoptosis, thus attenuating the pathological damage and increasing the survival rate in a mouse model of sepsis-induced acute kidney injury following cecal ligation puncture (CLP) [81]. On the other hand, Giangola et al. reported that rGas6 administration behaves as an anti-inflammatory agent capable of abrogating sepsis-induced organ dysfunction and neutrophil-induced acute lung injury (ALI), resulting in the amelioration of the overall survival in a mouse model of CLP-induced sepsis [82].

## 4. An Open Window on Sepsis

Sepsis is one of the most common life-threatening diseases widespread in the world [83]. A crucial point concerning sepsis is to reach a fast diagnosis because of the multiple comorbidities and underlying diseases presented by septic patients [84].

The sepsis definition, in use until 2016, was based on the host's inflammatory responses. Recently, physicians and researchers have begun to break up the pathophysiology of sepsis discovering that the host reaction to sepsis involves not only the inflammatory milieu but also a modification in nonimmunological pathways [85]. This latest understanding led to a review of the sepsis definition and, in 2016, the Sepsis-3 conference defined sepsis as a “life-threatening organ dysfunction caused by a deregulated host response to infection” and septic shock as a “subset of sepsis in which underlying circulatory and cellular/metabolic abnormalities are profound enough to substantially increase mortality” [86]. In this context, despite the presence of international recommendations [87], many points regarding the appropriate treatment still remain debatable [88–90]. As for the definition, diagnostic criteria have also changed and currently diagnosis is based on the detection of organ dysfunctions evaluated with the Sequential (Sepsis-Related) Organ Failure Assessment (SOFA) score.

In the past, the SOFA score was created with the aim of calculating the number and severity of the dysfunction in six organ systems (notably pulmonary, coagulation, hepatobiliary, cardiovascular, renal, and neurologic) [91]. The Sepsis-3 definitions also introduced a new diagnostic tool useful in the early identification of patients at risk of sepsis in the emergency department (ED): the quik-SOFA (qSOFA) [92].

Over the last decade, there has been great interest in finding out biomarkers that could improve both sepsis diagnosis and sepsis prognosis [93–95]. In 2017, Kim et al. demonstrated a possible prognostic utility of procalcitonin (PCT), presepsin (sCD14-subtypes), soluble suppression of tumorigenicity 2 (sST2), and Gal-3 in sepsis.

They suggested that a multimarker approach could be beneficial for an optimized management of patients with sepsis [93]. The idea of a multimarker approach has been recently reclaimed by Mearelli et al. in a multicenter prospective study comprising a large cohort of patients. They developed and validated a high-performing, reproducible, and cost-effective algorithm to assist physicians of the emergency department in distinguishing sepsis/septic shock from noninfectious systemic inflammatory response syndrome (SIRS) [96]. Nowadays, it is becoming evident that the use of biomarkers in clinical procedures can be helpful and essential for a correct diagnosis, to discriminate noninfectious SIRS, sepsis, and septic shock patients, and to estimate the prognosis.

The abovementioned Gal-3 is one of the novel Mer ligands identified by Caberoy et al. They showed that Gal-3 stimulates the phagocytosis of apoptotic cells and cellular debris through Mer activation [48]. Since Gal-3 is involved in efferocytosis and it was found significantly higher in patients with sepsis and septic shock, Ferreira et al. induced sepsis in both WT and Gal-3 knockout mice showing that the absence of Gal-3 was protective against sepsis. This phenomenon seems to be associated with the ability of Gal-3 to limit neutrophil migration to primary sites of infection, consequently favoring bacterial spreading and death [97].

The employment of TAM and their ligands as biomarkers in septic patients has already been described more than ten years ago. Borgel et al.'s and Gibot et al.'s groups were among the first to depict the correlation between Gas6 and sepsis condition in 2006 and 2007, respectively [98, 99]. Few years later, Ekman et al. confirmed that Gas6 levels are increased during sepsis [100], finding a correlation between Gas6 and the degree of organ damage. In addition, they showed an increase of sAxl as well, although without the same magnitude of Gas6. Indeed, Gas6 levels strongly correlated with IL-6 and PCT levels and the number of failing organs. Thus, Gas6 levels were associated with disease severity and organ dysfunction. New studies have been conducted on a cohort of septic patients diagnosed following the Sepsis-3 criteria [101, 102]. In a cohort of 129 patients, it was reported that Gas6 plasmatic levels at admission in an intensive care unit (ICU) were higher in nonsurvivors than survivors [101]. However, neither Gas6 nor sAxl levels investigated in this study were able to discriminate bacteremic from nonbacteremic patients or Gram-negative versus Gram-positive infections. Moreover, Gas6 was compared with well-known inflammatory/severity biomarkers and evidence was found for a correlation between Gas6 levels and IL-6, IL-8, IL-10, sAxl, and PCT levels. Gas6 and IL-8 were the only two biomarkers found to be differently expressed between survivors and nonsurvivors. Therefore, these two biomarkers seem to be able to predict mortality in septic/shock patients at the time of ICU admission. In the same study, Gas6 performed better than procalcitonin and C-reactive protein, which are broadly used to diagnose infection, even though Gas6 levels between survivors and nonsurvivors remained constant over time. According to these findings, Gas6 cannot predict sepsis evolution, unlike other inflammatory mediators, such as TNF-*α* and IL-1*β* [101]. The role of Gas6 in septic patients was recently highlighted also in sepsis-related acute lung injury (ALI) by Yeh et al. [102]. Indeed, ALI is one of the complications of sepsis, and it is known for its contribution to sudden deaths and morbidity [103]. In this study published in 2017, the authors enrolled 129 patients with a diagnosis of sepsis and they compared the patients with and without ALI, observing that Gas6 levels, together with IL-6 and IL-8 levels, were significantly elevated among patients who developed ALI. Since nowadays a prompt and correct ALI diagnosis is mandatory in order to develop an effective treatment, the authors suggested Gas6 as an early predictor of ALI. Moreover, they suggested that Gas6 could also improve the parameters of the lung injury prediction score, such as its discrimination and its positive and negative predictive values [102].

The role of Gas6 in inflammatory contexts seems to be mainly related to its interaction with Mer [104, 105]. Mer has a pivotal role in counterbalancing the proinflammatory effects of toll-like receptor 4 (TRL4) activation induced by LPS, as demonstrated by Lee et al. using an anti-Mer neutralizing antibody [104].

Natural occurring regulatory T cells (Tregs) play a central role in maintaining immunologic homeostasis and tolerance. Different studies reported an expansion in both percentage and number of Tregs along with an increase in their suppressive function during sepsis [106]. Heuer et al. showed that adoptive transfer of in vitro-stimulated Tregs was able to increase the survival and the bacterial clearance in a mouse model of CLP-induced polymicrobial sepsis [107]. Zhao et al. demonstrated that Tregs express both Mer and Axl and that Gas6 administration in vivo increases forkhead box P3 (Foxp3) expression and suppressive activity by CD4^+^CD25^+^ Tregs. In vitro stimulation of Tregs by Gas6 had no effects on IL-10 and transforming growth factor *β*1 (TGF-*β*1) production, but it increased Foxp3 and cytotoxic T-lymphocyte antigen 4 (CTLA-4) expression as well as the suppressive activity in a dose-dependent manner [108]. Hence, these studies suggest a possible role of Gas6 in tuning the immune response during sepsis by linking the innate and adaptive immune system.

However, the issue of comparing the response of the murine model of sepsis with human pathology is still open [109]. Regarding the focus of this review, we still know little about the response of TAM receptors and Gas6 in a murine sepsis model. Moreover, the levels of Gas6 and sAxl in both healthy and septic mice are not clear. Thus, the possibility that sAxl sequesters the endogenous circulating Gas6 is present in mice as well as in humans [45]. However, the administration of a large amount of exogenous Gas6 could overcome this problem by ameliorating the sepsis-induced multiorgan failure in septic mice, as recently demonstrated by Ni et al. [110]. Therefore, also in sepsis, where Gas6 levels are high, the injection of exogenous Gas6 seems to improve the outcome.

**Figure 2:**
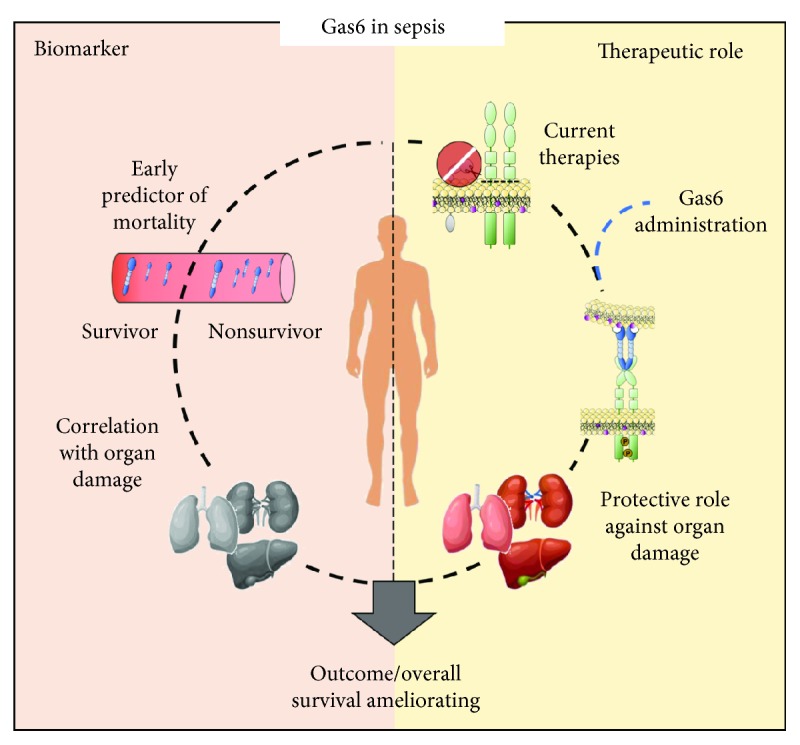
Gas6: the paradoxical role in sepsis. During sepsis, Gas6 could be used as an early biomarker in the routine management of septic patients since Gas6 plasma levels, measured at the time of ICU admission, can predict mortality and multiorgan failure. The high levels of Gas6 released in the bloodstream during sepsis seem to be aimed at counterbalancing sepsis dysfunctions; however, because inflammatory stimuli downregulate TAM receptors, the Gas6 overrelease is ineffective. Current therapy for sepsis is aimed at decreasing inflammatory stimuli. Gas6 administration after current therapy could operate on activated TAM receptors and protect the organs from sepsis-induced damage. The combination of a correct early diagnosis and the protective effects mediated by Gas6 could ameliorate the outcome/overall survival of patients.

Summarizing, on the basis of previous studies, it is possible to hypothesize the use of Gas6 as a biomarker in the complex pathophysiology of sepsis, since several data seem to suggest a role of Gas6 as a useful biomarker for discriminating between noninfectious SIRS, sepsis, and septic shock. Furthermore, Gas6 came out as an early predictor of mortality and was able to identify some life-threatening sepsis complications. Moreover, Gas6 administration could be envisaged as a therapeutic reinforcement to the current treatment, since it showed to be able to ameliorate the overall survival and to partially protect from the organ dysfunction in a mouse model of sepsis. In conclusion, the Gas6/TAM axis activation possibly ameliorates the tissue hypoperfusion, thus restoring the physiological tissue homeostasis and preserving organ function, with a positive impact on sepsis prognosis (Figure 2).

## Figures and Tables

**Table 1:** The widespread expression of the TAM receptor.

	Tyro3	Axl	Mer
Brain	(i) Microglial cells [4] (ii) Astrocytes [4]	(i) Microglial cells [4] (ii) Astrocytes [4]	(i) Microglial cells [4] (ii) Astrocytes [4]

Heart			(i) Cardiomyocytes [5]

Breast			(i) Mammary epithelial cells [6]

Lung		(i) Macrophages CD11bl^ow^CD11c^high^ [7]	(i) *Alveolar macrophages* [8]

Liver	(i) *Kupffer cells* [9]	(i) *Kupffer cells* [9] (ii) *HSCs (q/a)* [9] (iii) *LSECs* [9] (iv) *Hepatocytes* [9]	(i) *Kupffer cells* [9] (ii) *HSC (a)* [9] (iii) *LSEC* [9]

Spleen		(i) DCs CD11c^high^ [10]	(i) Macrophages F4/80^high^, B220^–^, CD11c^+^ and MHCII^+^ red pulp [11] (ii) Macrophages F4/80^+^CD68^+^ (tingible body) [11]

Kidney	(i) *Podocytes* [12]		(i) *Podocytes* [12]

Testis	(i) Sertoli cells [13]	(i) Sertoli cells [13]	(i) Sertoli cells^low^ [13] (ii) Leydig cells [13]

Peritoneum		(i) Macrophages [14]	(i) Macrophages [14]

Blood/BM derived	(i) *Platelets* [15] (ii) Monocytes^low^ (iii) Monocyte-derived macrophages^low^ [16] (iv) NK cells [17] (v) DC CD11c^+^ [18]	(i) *Platelets* [15] (ii) Monocytes^high^ (iii) Monocyte-derived macrophages^low^ [16] (iv) NK cells [17] (v) DC CD11c^+^ [18]	(i) *Platelets* [15] (ii) Monocytes^low^ (iii) Monocyte-derived macrophages^high^ [16] (iv) NK cells [17] (v) DC CD11c^+^ [18] (vi) DCs CD11b^+^ and B220^+^ [19] (vii) NKT cells [20]

Italic shows TAM expression located in human cells; all the others were found in murine cells. BM derived: bone marrow derived; HSCs: hepatic stellate cells; LSECs: liver sinusoidal endothelial cells; DCs: dendritic cells; NK: natural killer; NKT: natural killer T.

## Abbreviations

Gas6: Growth arrest-specific 6
RTKs: Tyrosine kinase receptors
DCs: Dendritic cells
BM: Bone marrow
HSCs: Hepatic stellate cells
LSECs: Liver sinusoidal endothelial cells
NK: Natural killer
NKT: Natural killer T
Pros1: Protein S
SHGB: Sex hormone-binding globulin
Gla: Gamma-carboxyglutamic acid rich
PtdSer: Phosphatidylserine
PI3K: Phosphoinositide 3 kinase
MAP: Mitogen-activated protein
NF-*κ*B: Nuclear factor-light-chain-enhancer of activated B cells
STAT: Signal transducer and activator of transcription protein
PLC: Phospholipase C
Grb2: Growth factor receptor-bound protein 2
ERK: Extracellular-signal-regulated kinase
SOCS: Suppressor of cytokine signaling proteins
TKO: Triple knockout
WT: Wild type
SLE: Systemic lupus erythematosus
APCs: Antigen-presenting cells
IL: Interleukin
ADAM17: A disintegrin and metalloproteinase domain 17
TACE: Tumor necrosis factor-alpha converting enzyme
PRRs: Pattern-recognition receptors
LPS: Lipopolysaccharide
Poly:C: Polycytidylic acid
PolyI:C: Polyinosinic:polycytidylic acid
sAxl: Soluble Axl
sMer: Soluble Mer
TK: Tyrosine kinase
FN III: Fibronectin type III domains
CKD: Chronic kidney disease
CSF: Cerebrospinal fluid
AD: Alzheimer's disease
CIDP: Chronic inflammatory demyelinating polyneuropathy
I/R: Ischemia/reperfusion
TNF-*α*: Tumor necrosis factor *α*
ALT: Alanine aminotransferase
AST: Aspartate aminotransferase
rGas6: Recombinant Gas6
CLP: Cecal ligation puncture
SOFA: Sequential (sepsis-related) organ failure assessment
ED: Emergency department
qSOFA: Quick SOFA
PCT: Procalcitonin
Gal-3: Galectin-3
sST2: Soluble suppression of tumorigenicity 2
SIRS: Systemic inflammatory response syndrome
ALI: Acute lung injury
TLR4: Toll-like receptor 4
TSLP: Thymic stromal lymphopoietin
TSLPR: TSLP receptor
CCL17: Chemokine (C-C motif) ligand 17
Tregs: Regulatory T cells
Foxp3: Forkhead box P3.
